# Improving vagal activity ameliorates cardiac fibrosis induced by angiotensin II: *in vivo* and *in vitro*

**DOI:** 10.1038/srep17108

**Published:** 2015-11-24

**Authors:** Jin-Jun Liu, Ning Huang, Yi Lu, Mei Zhao, Xiao-Jiang Yu, Yang Yang, Yong-hua Yang, Wei-Jin Zang

**Affiliations:** 1 Department of Pharmacology, Xi’an Jiaotong University Health Science Center, Xi’an, Shaanxi, P. R. China

## Abstract

Cardiac remodeling is characterized by overactivity of the renin–angiotensin system (RAS) and withdrawal of vagal activity. We hypothesized that improving vagal activity could attenuate cardiac fibrosis induced by angiotensin II (Ang II) *in vivo* and *in vitro*. Rats were subjected to abdominal aorta constriction (AAC) with or without pyridostigmine (PYR) (31 mg/kg/d). After 8 weeks, PYR significantly decreased Ang II level, AT1 protein expression, and collagen deposition in cardiac tissue and improved heart rate variability, baroreflex sensitivity and cardiac function, which were abolished by atropine. *In vitro*, treatment of cardiac fibroblasts (CFs) with Ang II (10^−7^ M) increased cell proliferation, migration, transformation, and secretory properties, which were significantly diminished by acetylcholine (ACh, 10^−6^ M). Subsequently, Ang II significantly increased collagen type I expression as well as metalloproteinase (MMP)-2 expression and activity. Transforming growth factor (TGF)-β1 expression and Smad3 phosphorylation presented a similar trend. Notably, the knockdown of the acetylcholine M_2_ receptor by siRNA could abolish ACh anti-fibrotic action. These data implicated cholinesterase inhibitor can increase vagal activity and reduce local Ang II level, and ACh inhibit Ang II pro-fibrotic effects. Our findings suggested that the parasympathetic nervous system can serve as a promising target for cardiac remodeling treatment.

Cardiac remodeling, characterized by left ventricular (LV) hypertrophy, cardiac inflammation, and fibrosis, is a major risk factor for cardiovascular morbidity and mortality and a leading cause of chronic heart failure. It is generally agreed that activation of the renin-angiotensin system (RAS) plays an important pathophysiological role in cardiac remodeling^1^. Cumulative evidence suggests that cardiac RAS is significantly activated in cardiac remodeling induced by pressure overload^2^. Yamamoto *et al.* recently demonstrated that both serum and cardiac tissue levels of angiotensin II (Ang II) increased in a rat model with transverse aortic constriction^3^. Indeed, long-term infusion of Ang II, using a minipump, is associated with persistent hypertension, myocardial hypertrophy, fibrosis, and adverse remodeling, which, if untreated, can progress to heart failure^4^. Ang II inhibition with either angiotensin-converting enzyme inhibitors or angiotensin II type 1 receptor (AT1R) blockers significantly improves cardiac function and induces the regression of cardiac remodeling in patients with hypertension and in animal models after myocardial infarction^5^.

Besides RAS, the cardiac autonomic nervous system (ANS) imbalance is intimately associated with the pathogenesis of cardiovascular disease (CVD)^6^,^7^. Considerable evidence shows that the vagal nerve function is attenuated in a variety of cardiovascular diseases such as hypertension, heart failure, and diabetes. Vagal control is abnormal in heart failure, occurring at early stages of LV dysfunction. This reduced vagal function is associated with worse outcomes in patients with heart failure^8^. It has also been demonstrated that suppressed vagal activity accelerates cardiac remodeling and increases the risk of developing life-threatening tachyarrhythmia. In contrast, improving vagal activity is thought to be a promising strategy for CVD. Previous studies showed that electrical stimulation of the vagal nerve improves cardiac function in rats and dogs with heart failure^9^,^10^. Moreover, rodent studies using cholinesterase blockers, e.g., donepezil and pyridostigmine (PYR), described an attenuation of both cardiac remodeling and cardiac dysfunction progression as well as improvement of the long-term survival of animals with heart failure^11^,^12^. However, there are no substantial studies to assess whether improving vagal activity can inhibit abdominal aorta constriction (AAC)-induced cardiac remodeling and RAS activation.

It is well known that, during cardiac remodeling, cardiac fibroblasts (CFs) play a central role in the maintenance of the extracellular matrix (ECM) and undergo hyperplasia in response to some humoral factors such as Ang II and endothelin^13^,^14^. Increasing evidence suggested that Ang II, via AT1R, upregulates the expression of matrix metalloproteinases (MMPs) and increases fibroblasts migration, proliferation, ECM deposition, fibrosis, and adverse remodeling^15^. It has been shown that, in neonatal rat cardiomyocytes, acetylcholine (ACh), acting through M_2_ receptors, activates nitric oxide synthesis, exerting anti-hypertrophic effects^16^. It is noted that enhancing ACh signaling may prevent cardiomyocyte hypertrophy and cardiac remodeling^17^. Recent studies in our laboratory demonstrated that ACh improves contractile function and prevents Ang II-induced H9c2 cells apoptosis and oxidative stress, consolidating the cardiovascular therapeutic benefit of ACh^18^. However, there are no reports evaluating the effects of ACh on Ang II-induced CFs activation. In this study, we investigated whether an acetylcholinesterase inhibitor ameliorates cardiac remodeling induced by pressure overload by inhibiting RAS activation and whether ACh cardioprotective benefits are related, in part, to the suppression of Ang II-induced CFs activation.

## Results

### PYR reverses AAC-induced cardiac fibrosis and improve cardiac function

**Figure 1:**
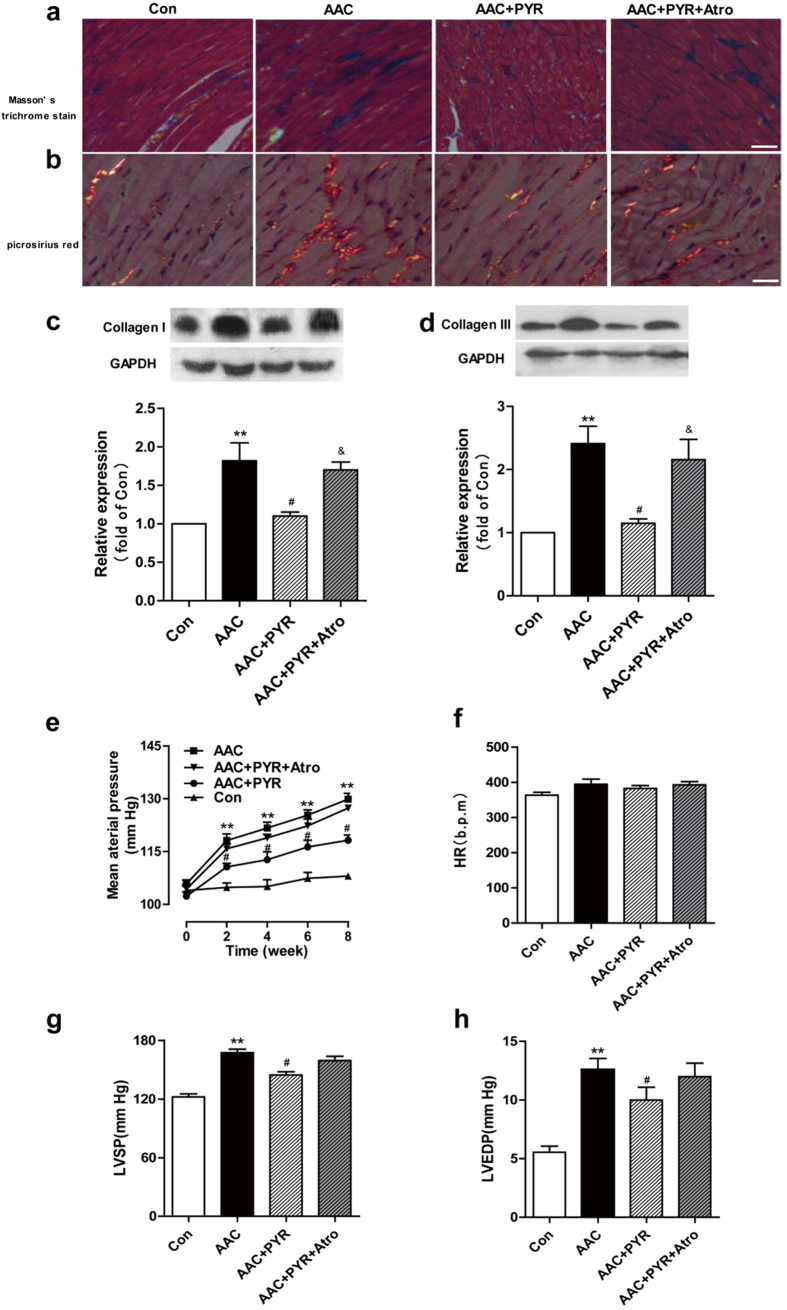
PYR inhibits cardiac fibrosis and improves cardiac function. (**a**) The image shows Masson’s trichrome stained collagen in the cardiac interstitium. Blue parts represent collagen. Bar = 50 μm. (**b**) Representative LV sections stained with picrosirius red for collagen type I (red) and III (orange yellow) from all groups as indicated. Bar = 25 μm. (**c**,**d**) LV tissues were analyzed for collagen type I and III by western blot. (**e**) MAP, mean arterial pressure. (**e**) HR, heart rate. (**f**) LVSP, left ventricular systolic pressure. (**h**) LVEDP, left ventricular end-diastolic pressure. Data are presented as means ± SEM. *P < 0.05, **P < 0.01 *vs.* control group; ^#^*P* < 0.05 *vs.* AAC group; ^&^*P* < 0.05 *vs.* PYR group.

To determine if PYR plays a vital role in cardiac fibrosis induced by AAC, we treated AAC-operated rats with PYR or with normal saline for 8 weeks after surgery. Masson’s results showed that PYR significantly attenuated collagen deposition induced by AAC (blue area). Treatment with atropine abolished PYR’s effects on collagen deposition (Fig. 1a). Picrosirius red under polarized light showed a marked increase in collagen type I (red) and III (orange yellow) for AAC compared with control. PYR reversed this deposition and the effects of PYR was inhibited by atropine (Fig. 1b). Moreover, the expression of the ECM protein collagen type I and III were enhanced in AAC group. Co-treament with PYR reverses these changes, and atropine abolished the protective effect of PYR (Fig. 1c,d).

We monitored hemodynamic parameters and found that there are no significant differences in heart rate (HR) in all experimental groups. Mean arterial pressure (MAP), left ventricular systolic pressure (LVSP) and left ventricular end diastolic pressure (LVEDP) after ACC-operated were significantly improved by PYR. Atropine administration abolished these changes (Fig. 1e–h).

### PYR decreases Ang II level and improves vagal activity

**Figure 2:**
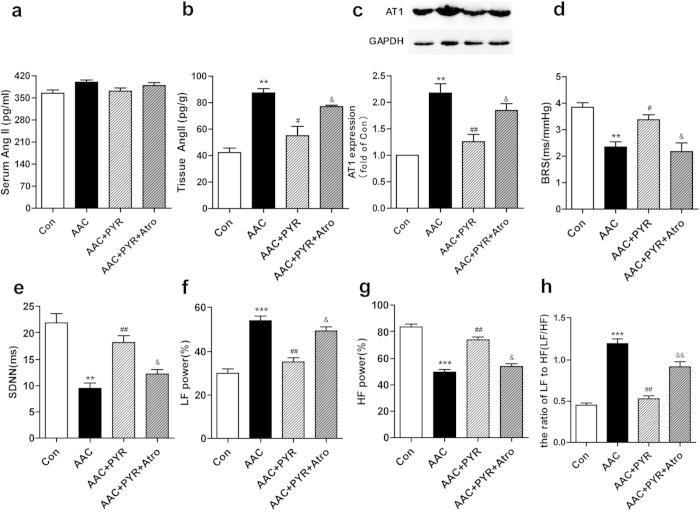
PYR decreases Ang II level and improves vagal activity. (**a**) Radioimmunoassay was performed to detect Ang II in serum and (**b**) left ventricular tissues. (**c**) Western blot and quantitative evaluation of AT1 expression. (**d**) Baroreflex sensitivity (BRS). (**e**) Standard deviation of RR Interval (SDNN). (**f**) Normalized low frequency power (LF power; %). (**g**) Normalized high frequency power (HF power, %). (**h**) Absolute values of low/high frequency power ratio (LF/HF). Data are presented as means ± SEM. **P < 0.01 and ***P < 0.001 *vs*. control group; ^#^*P* < 0.05 and ^# #^*P* < 0.01 *vs*. AAC group; ^&^*P* < 0.05 and ^&&^*P* < 0.01 *vs*. PYR group.

RAS activation, with Ang II synthesis and release, plays an important pathophysiological role in cardiac remodeling. Thus, we determine whether PYR protective effect was due to an inhibition of Ang II generation. There were no significant differences in serum Ang II level in all experimental groups (Fig. 2a). Compared with control group, Ang II and AT1 expression in left ventricular tissues was significantly increased in AAC group. PYR treatment significantly decreased Ang II level and AT1 expression (Fig. 2b,c). As shown in Fig. 2d, Baroreflex sensitivity (BRS) was dramatically decreased in the AAC group when compared with the control group, while BRS improvement was observed in the AAC+PYR group. In the frequency domain, LF power was lower and HF power was higher in the PYR rats. The LF/HF ratio was lower in the PYR group. In the time frequency, standard deviation of normal-to-normal (SDNN) was decreased in the AAC group compared with the control group, and PYR normalized these parameters in the AAC rats (Fig. 2e–h). Taken together, Heart rate variability (HRV) data indicated that PYR treatment enhanced vagal control and autonomic balance. PYR effects were inhibited by atropine.

### ACh inhibits Ang II-induced collagen production

**Figure 3:**
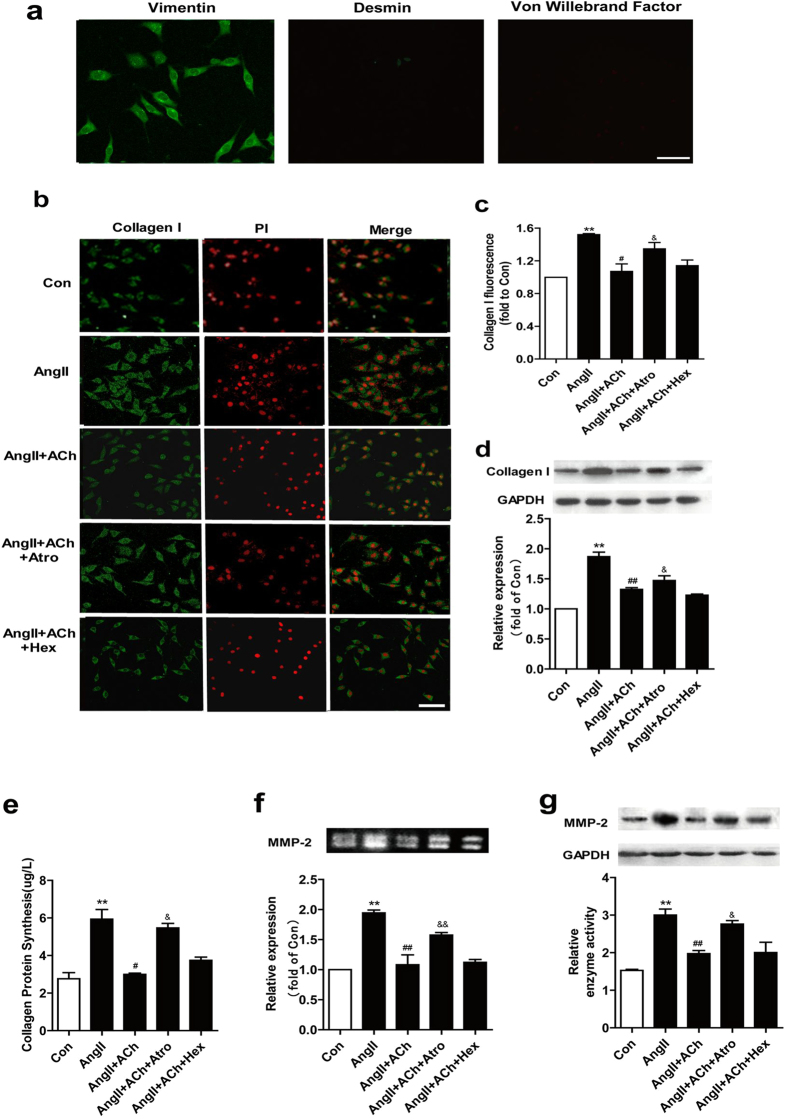
ACh attenuates Ang II-induced collagen production. (**a**) Fluorescence micrographs of vimentin, Von Willebrand Factor and desmin. Bar = 25 μm. (**b,c**) For collagen type I assay, CFs were cultured in the indicated experimental conditions and immunostained with an antibody against collagen I (green), and the histograms showed the corresponding densitometric analyses of the intensity of collagen I fluorescence signal. Bar = 50 μm. (**d**) Western blot analysis of collagen I expression in CFs. The densitometric analysis of the bands normalized to GAPDH is reported in the histograms. (**e**) The content of hydroxyproline was measured by using a bioluminescent assay. (**f**) MMP-2 activity was measured by gelatin zymography. (**g**) Western blot and quantitative evaluation of MMP-2 expression. Data are presented as means ± SEM. **P < 0.01 *vs*. control group; ^#^*P* < 0.05 and ^# #^*P* < 0.01 *vs*. Ang II group; ^&^*P* < 0.05 and ^&&^*P* < 0.01*vs*. ACh group.

Vimentin, Von Willebrand Factor and desmin were used to identification of cardiac fibroblasts (CFs). The results showed that the composition of the cultures was >98% vimentin positive, desmin negative, and Von Willebrand Factor negative (Fig. 3a).

As mentioned above, collagen deposition is an important pathological phenomenon during cardiac remodeling. To determine whether ACh prevents Ang II-induced collagen synthesis, cells were co-treated with atropine (10^−5^ M) or hexamethonium (10^−5^ M) and ACh (10^−6^ M) and Ang II (10^−7^ M). Compared with control, CFs treated with Ang II showed an increase in collagen I production. ACh ameliorated Ang II-induced collagen I generation (Fig. 3b,c). Western blot analysis also demonstrated that ACh prevented collagen I expression (Fig. 3d). The increase in hydroxyproline content induced by Ang II was significantly reduced after ACh treatment (Fig. 3e). All effects of ACh were inhibited by atropine. Because collagen accumulation depends not only on its production, but also on its MMP-mediated degradation, we examined MMP-2 expression and activity. As shown in Fig. 3f,g, MMP-2 expression and activity were significantly enhanced by Ang II. These effects were inhibited by ACh. In addition, cells treated with atropine rather than hexamethonium displayed lower MMP-2 expression and activity compared with that of ACh-treated cells.

### ACh suppresses Ang II-induced viability and proliferation of CFs

**Figure 4:**
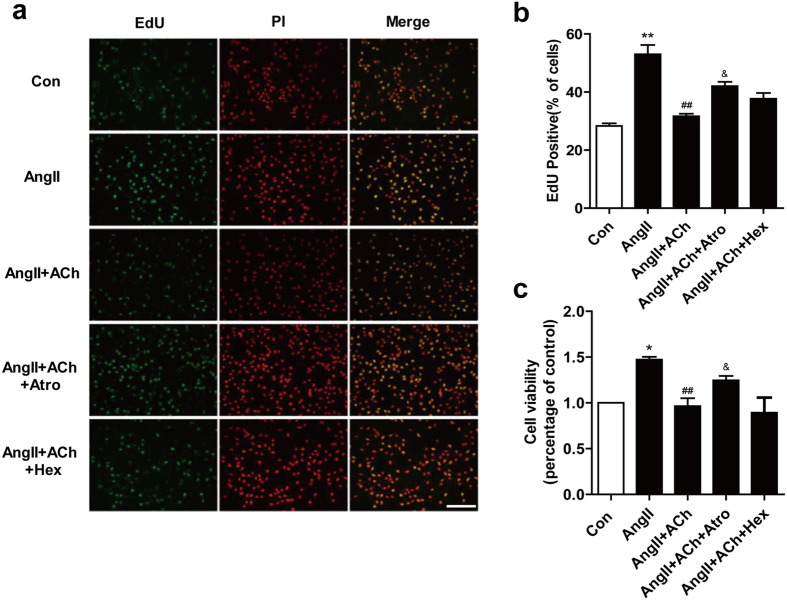
ACh suppresses Ang II-induced proliferation and viability. (**a**) Representative photomicrographs of 5-ethynil-2-deoxyuridine (EdU) staining (left panel) and corresponding total cell photomicrographs (middle panel). Proliferative cell nuclei were labeled with EdU (green). Cell nuclei were labeled by using PI (Red). (**b**) Effect of ACh on cell proliferation as determined by EdU staining. Quantitative data showing the percentage of EdU-positive cells (number of green *vs.* red nuclei). Bar = 50 μm. Data are presented as means ± SEM. *P < 0.05 and **P < 0.01 *vs*. control group; ^##^*P* < 0.01 *vs*. Ang II group; ^&^*P* < 0.05 *vs.* ACh group.

CFs are a major non-muscle cell type responsible for ECM regulation and remodeling, with fibroblasts proliferation playing an important role in the remodeling process. In order to investigate whether ACh protective effect was due to an inhibition of cell proliferation and viability we did the following experiments. As shown in Fig. 4, in Ang II treated cells, EdU incorporation and cell viability obviously increased. ACh significantly attenuated the hyperplasia and viability of CFs induced by Ang II. The protective effect of ACh was blocked by atropine, while co-treatment with hexamethonium showed no difference compared to that in the ACh group.

### ACh reduces Ang II-induced cells transformation and migration

**Figure 5:**
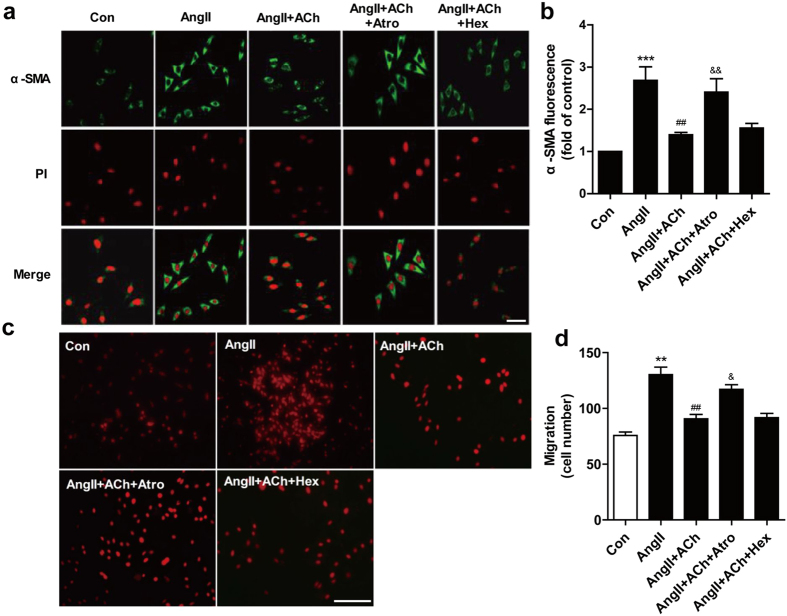
ACh inhibits CFs migration and transformation induced by Ang II. (**a,b**) Fluorescence immunohistochemistry using a specific α-SMA first antibody following by a second antibody conjugated to FITC was performed to demonstrate fibroblast transformation induced by Ang II. Nuclei were stained with PI. Bar = 25 μm. (**c,d**) Representative pictures of cell migration detected by transwell migration assay. Bar = 50 μm. Data are presented as means ± SEM. **P < 0.01 and ***P < 0.001 *vs*. control; ^##^*P* < 0.001 *vs.* Ang II; ^&^*P* < 0.05 and ^&&^*P* < 0.01 *vs*. ACh.

The expression of α-SMA is a hallmark of fibroblast differentiation. Therefore, CFs were pretreated with ACh for 30 min before Ang II administration and α-SMA expression was assessed. As shown in Fig. 5a, Ang II stimulation increased α-SMA intensity and organization. ACh effectively reduced Ang II effect. ACh inhibitory effects on Ang II-induced migration of CFs were examined using a transwell assay and representative pictures are shown (Fig. 5c). Incubation with Ang II significantly upregulated cell migration compared to control. Treatment with ACh significantly reversed the increase of cell migration induced by Ang II. The results indicated that atropine but not hexamethonium blocked ACh protective effect.

### ACh inhibits TGF-β1/Smad3 pathway activation induced by Ang II

**Figure 6:**
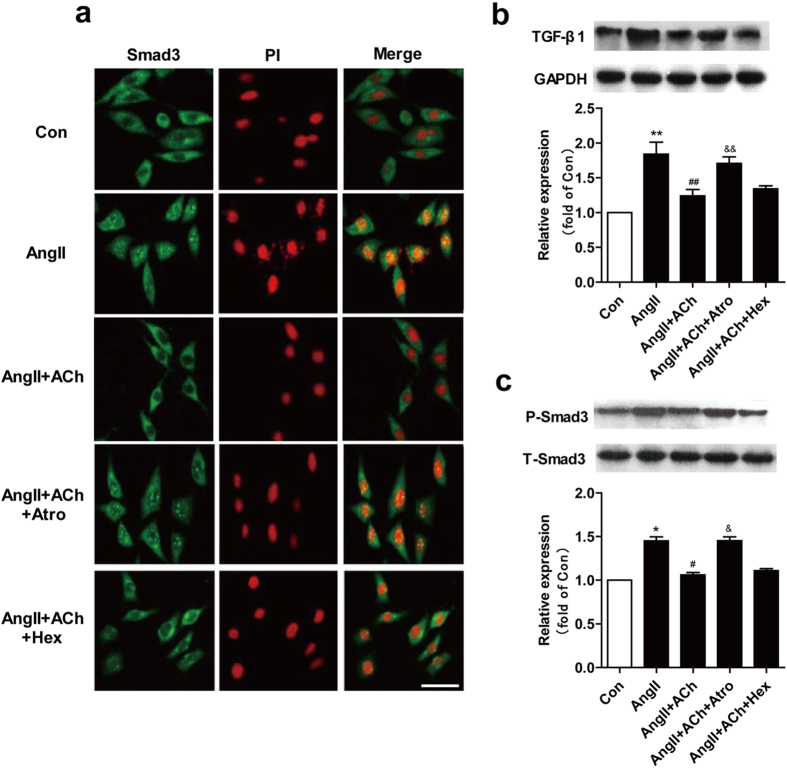
ACh inhibits the Ang II-induced activation of the TGF-β1/Smad3 pathway. (**a**) Immunofluroescence images show that ACh blocks Smad3 nuclear translocation induced by Ang II. (**b**,**c**) Western blot evaluated expression of TGF-β1 and P-Smad3. Bar = 25 μm. Data are presented as means ± SEM. *P < 0.05 and **P < 0.01 *vs*. control; ^#^*P* < 0.05 and ^##^*P* < 0.01 *vs*. Ang II; ^&^*P* < 0.05 and ^&&^*P* < 0.01 *vs*. ACh.

To investigate the mechanism involved in the attenuation of Ang II-induced cardiac fibrosis by ACh, we measured Transforming growth factor (TGF)-β1 expression. Compared with control, Ang II treated cells showed an increase in TGF-β1 expression. However, pretreatment of the cells with ACh reversed this change (Fig. 6b). Western blot analysis demonstrated that Ang II treatment significantly increased phosphorylated Smad3 level. ACh pretreatment inhibited this process. Both ACh effects were inhibited by atropine (Fig. 6c). Smad3 nuclear translocation is one of the key components modifying the TGF-β1/Smad3 pathway. Immunofluorescent analysis was used to assess whether ACh blocks Smad3 nuclear translocation. As shown in Fig. 6a, Ang II treatment greatly stimulated Smad3 nuclear translocation as indicated by strong Smad3 staining in the nucleus. ACh pretreatment inhibited Ang II-induced Smad3 translocation. However, ACh effects were inhibited by atropine rather than hexamethonium.

### M_2_ AChR knockdown abrogates ACh anti-fibrotic effect

**Figure 7:**
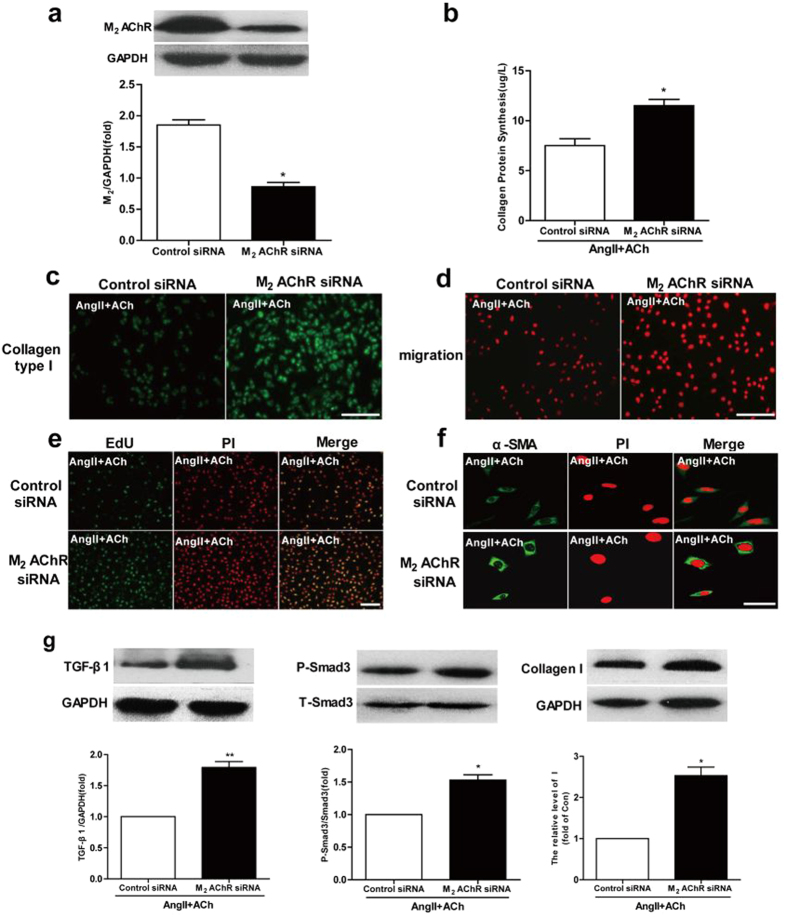
M_2_ AChR knockdown abrogates ACh anti-fibrotic effect. (**a**) Western blot was performed to assess M_2_ AChR knockdown. (**b**) Hydroxyproline content was measured by using a bioluminescent assay. (**c**) M_2_ AChR siRNA transfection increases collagen I contents in the presence of ACh in Ang II treated cells. Bar = 50 μm. (**d**) M_2_ AChR siRNA transfection increases fibroblasts migration. Bar = 50 μm. (**e**) Cell proliferation was measured as the percentage of EdU-positive cells. Bar = 50 μm. (**f**) M_2_ AChR siRNA transfection increases fibroblast transformation. Bar = 25 μm. (**g**) Expression of TGF-β1, P-Smad3, and collagen I. Data are presented as means ± SEM. *P < 0.05 and **P < 0.01 *vs*. control siRNA group.

To investigate the role of M_2_ AChR in collagen production, CFs were transfected with M_2_ AChR or control siRNA and exposed to Ang II in presence of ACh 48 h after transfection. As shown in Fig. 7a, compared to the control siRNA group, M_2_ AChR siRNA reduced M_2_ AChR expression levels by 51%. Our results showed that compared with the control siRNA group, transfection of M_2_ AChR siRNA markedly increased the hydroxyproline content, collagen I production, and EdU incorporation, cell transformation and migration (Fig. 7b–f). Besides, TGF-β1, P-Smad3, and collagen I expression was also significantly higher in the M_2_ AChR siRNA group than that of the control siRNA group (Fig. 7g). These findings support that M_2_ AChR plays a predominant role in ACh-mediated inhibition of collagen production.

## Discussion

The key findings of the present study were: (i) PYR, a cholinesterase inhibitor, protects against cardiac remodeling induced by pressure overload via inhibition of RAS activation; (ii) ACh suppresses Ang II-induced cell proliferation, migration, and transformation as well as collagen synthesis, partially through the TGF-β1/Smad3 signaling pathway; (iii) M_2_ AChR plays a pivotal role in ACh anti-fibrotic action in CFs. These findings suggest that pharmacological restoration of vagal tone may be a potential therapeutic strategy for cardiac remodeling treatment.

Growing evidence showed that RAS overactivity and vagus nerve withdrawal are typically associated with many cardiovascular diseases^19^. Previous studies demonstrated that RAS activation plays a vital role in AAC-induced cardiac remodeling^20^. Inhibition of Ang II generation using angiotensin-converting enzyme inhibitors or blockade of AT1R by competitive receptor antagonists is clinically used to improve hemodynamic parameters and to inhibit adverse cardiac remodeling in patients after myocardial infarction^5^. Cumulative evidence suggest that Reduced vagal activity is associated with increased mortality in patients with chronic heart failure and further vagal withdrawal precedes acute decompensation^21^,^22^, and increased vagal nerve activity significantly improve left ventricular hemodynamics, markedly reduce MI size and decrease mortality^10^. We previously showed that PYR ameliorates cardiac remodeling induced by myocardial infarction via inhibition of the TGF-β1-activated kinase pathway^23^. PYR administration have systemic effects including increasing vagal tone, reducing sympathetic tone, and increased vascular endothelial growth factor^24^,^25^,^26^. Recent studies demonstrated that PYR treatment prevents isoproterenol-induced autonomic dysfunction and inhibit cardiac remodeling^26^.

In the present study, we demonstrated that PYR could restore vagal tone, improve cardiac function and attenuate cardiac remodeling after AAC operation. However, the effects of PYR were blocked by atropine (Fig. 1). We also found that PYR administration decreased cardiac tissue Ang II concentration and AT1 expression (Fig. 2). These results indicate that improving vagal activity can inhibit AAC-induced cardiac remodeling via suppressing local RAS activation. However, it is not clear whether PYR can only decrease Ang II level or directly antagonize Ang II pro-fibrotic. To investigate the mechanism for PYR anti-fibrotic effect, we evaluated the effect of ACh on collagen synthesis and activation in fibroblasts induced by Ang II.

As a key source of ECM components, fibroblasts are the most prevalent cell type and are responsible for regulating normal myocardial function. They also play a critical role in the adverse myocardial remodeling that occurs with hypertension, myocardial infarction, and heart failure^4^,^27^. Studies in rat and human CFs showed that Ang II via AT1 activation mediates fibroblast proliferation and collagen production^28^. Recent reports showed that ACh presents potential beneficial effects on cardiovascular diseases^29^. Importantly, our previous study showed that ACh attenuated Ang II-induced apoptosis by suppressing oxidative stress-mediated mitogen-activated protein kinase activation^30^. Based on previous study, we hypothesize that ACh possesses an anti-fibrotic effect in Ang II stimulated fibroblasts by suppressing collagen synthesis. The results from our current study showed that Ang II increased intracellular collagen I production (Fig. 3b–d). In addition, we found that ACh inhibited the increase in hydroxyproline content induced by Ang II (Fig. 3e). Collagen deposition depends not only on its production, but also on its degradation, which relies on enzymes such as MMPs. It is of note that MMP-2 expression also increases in rat fibroblast exposed to anoxia-reoxygenation. We conducted studies to determine MMP-2 expression and activity in CFs. As shown in Fig. 3f,g, Ang II increased MMP-2 expression and activity, which were effectively abolished by ACh pretreatment. Atropine could block the effect of ACh, while hexamethonium did not.

A growing body of evidence indicated that many of the functional effects of fibroblasts are mediated through transformation to myofibroblasts and increased proliferative, migratory, and secretory properties^31^. Fibroblasts proliferation plays a vital role in the development of cardiac remodeling. Thus, it is likely that, in the current study, Ang II stimulated cell proliferation and viability, resulting in an increase in CFs proliferation. This increase in proliferation could be blocked by ACh pretreatment (Fig. 4). Fibroblasts activation is characterized by the expression of α-SMA, which is an important phenotypic marker for myofibroblasts. It has been suggested that α-SMA expression increases significantly in myofibroblasts in hypertrophic and fibrotic hearts^32^. Consistent with the results of these reports, our present study showed that Ang II stimulation greatly increased α-SMA production. Interestingly, ACh pretreatment significantly reduced Ang II-induced α-SMA expression in CFs (Fig. 5a). To the best of our knowledge, it is the first report that shows that ACh exerts its cardioprotective properties by blocking fibroblasts differentiation to myofibroblasts. Fibroblasts represent the major non-muscle cell type in the heart responsible for cardiac remodeling, with migration of fibroblasts into injured tissues playing a significant role in the remodeling process. Our data revealed that ACh could decrease cell migration induced by Ang II (Fig. 5b). This finding is in agreement with previous reports showing that forced expression of reversion-inducing-cysteine-rich protein with Kazal motifs attenuates Ang II-induced fibroblasts migration^33^.

As an important fibrotic factor, TGF-β1 expression significantly increases in different pathological settings and cell types^34^. A large body of evidence suggests that TGF-β1 promotes the synthesis of ECM components and that the development of cardiac fibrosis is controlled by a regulatory network involving Ang II^35^. Li *et al.* found that an anti-TGF-β1 antibody attenuated Ang II-induced periostin expression^36^. Consistent with previous results, we observed that Ang II increased TGF-β1 expression (Fig. 6b). Smad3 proteins are essential components of the intracellular signaling pathway utilized by TGF-β1 and participate in TGF-β1-induced fibrosis^37^. Studies revealed that Smad3 plays a pivotal role in mediating TGF-β1-induced stimulation of collagen gene expression in normal fibroblasts^38^,^39^. In the present study, ACh inhibited Ang II-induced phosphorylation of Smad3 and blocked Smad3 nuclear translocation (Fig. 6a,c). These results provide a new ACh therapeutic target in cardiac remodeling.

It should be noted that ACh cardioprotective action is mediated through binding to its receptor, either muscarinic (M) AChR or nicotinic (N) AChR. Convincing evidence showed that, in endothelial cells, a7-nAChR is strongly involved in angiogenesis^39^,^40^. On the other hand, numerous studies have demonstrated that ACh-mediated signaling suppresses myocardial oxidative stress and remodeling via M AChR^41^. In the present study, our results demonstrated that atropine, rather than hexamethonium, abolished ACh cardioprotective effect. Importantly, M_2_ AChR knockdown by siRNA reversed ACh anti-fibrotic effect, indicating that ACh anti-fibrotic effect is mediated mainly via the M_2_ AChR (Fig. 7).

In conclusion, the present study demonstrates that improving vagal activity can inhibit cardiac remodeling induced by pressure overload. Cholinesterase inhibitor can suppress local RAS activation and decrease Ang II level. ACh can ameliorate Ang II-induced fibrosis via M_2_ AChR in CFs, which may involve the activation of the TGF-β1/Smad3 signaling pathway. These findings support the idea that the parasympathetic nervous system can serve as a promising target for the treatment of cardiac remodeling.

## Materials and Methods

### Materials

Dulbecco’s Modified Eagle’s Medium (DMEM) and fetal bovine serum (FBS) were purchased from Thermo Fisher (Waltham, MA, USA). Ang II, ACh, atropine, penicillin, streptomycin, 3-(4,5-dimethylthiazol-2-yl)-2, 5-diphenyl tetrazolium bromide (MTT), DMSO, and propidium iodide (PI) were obtained from Sigma-Aldrich (Saint Louis, MI, USA). Anti-Vimentin, Anti-Desmin, anti-GAPDH, anti-TGF-β1, anti-Smad3, anti-P-Smad3, and anti-MMP-2 were purchased from Cell Signaling Technology (Danvers, MA, USA). Anti-Von Willebrand Factor, Anti-I collagen, Anti-III collagen, anti-α-SMA and fluorescein-isothiocyanate-conjugated (FITC) anti-rabbit second antibody were purchased from Abcam (Cambridge, UK). 680RD Conjugated Goat polyclonal Anti-Mouse IgG and 800CW Conjugated Goat polyclonal Anti-Rabbit IgG were purchased from LiCor Biosciences (Lincoln, NE, USA). Collagenase II was from Worthington (Columbia, NJ, USA). PYR was obtained from Shanghai ZhongxiSunve Pharmaceutical (Co, Ltd, Shanghai, China).

### Animals, surgery, and study design

Our experimental procedures were in accordance with the Guidelines on the Care and Use of Laboratory Animals (National Institutes of Health publication no. 85-23, revised 1996) and were approved by the Ethics Committee of Xi’an Jiaotong University. Adult male Sprague-Dawley rats (8–10 weeks old) were provided by the Experimental Animal Centre of Xi’an Jiaotong University. Abdominal aorta constriction was performed, as described previously^42^. Briefly, rats were anesthetized with pentobarbital sodium (45 mg/kg) by intraperitoneal injection, and the aorta was dissected above the two renal arteries. A silver clip (0.70 mm internal diameter) was placed on the aorta abdominalis above the level of the left renal arteries. Three days afteraortic constriction, rats were randomly divided into four groups: AAC group (AAC, n = 7), PYR+AAC (PYR, 31 mg/kg/day, i.g., n = 8), PYR+AAC+atropine (Atro, 0.6 mg/kg/day, i.p., n = 8), and the sham group was identical, except for the clip placement. At day 56, the animals were euthanized, hemodynamic parameters were recorded, and hearts were excised for further investigation.

### Cell culture and study design

Adult cardiac fibroblasts (CFs) were isolated from male Sprague-Dawley rats (8–10 weeks old). The ventricles were minced and digested in DMEM containing 0.01% collagenase II at 37 °C for 30 minutes. Cells were collected and plated for 1 h at 37 °C. The cultured CFs were fixed with 4% paraformaldehyde 20 min at room temperature, and permeabilized by incubating for 15 min in 0.01% Tween 20 in PBS, and then were incubated with antibodies to vimentin (1:100), desmin (1:100) and Von Willebrand Factor(1:100), followed by incubation with a FITC anti-rabbit second antibody (1:200). Images were acquired by confocal microscopy. The viability of cells were evaluated using the MTT assay^43^. The CFs were cultured in DMEM supplemented with 10% (v/v) FBS (Thermo fisher), 100 U/mL penicillin (Sigma-Aldrich) and 100 μg/mL streptomycin (Sigma-Aldrich). Cells were starved in serum-free DMEM for 6 h and then treated with or without Ang II (10^−7^ M), ACh (10^−6^ M), atropine (10^−5^ M, a non-selective muscarinic receptor antagonist), or hexamethonium (10^−5^ M, a non-selective nicotinic receptor antagonist). The incubation was continued for another 24 h, and the cells were then harvested and extracted for analysis.

### Cardiac function assay

At day 56, animals were anesthetized with sodium pentobarbital for hemodynamic studies. Right carotid arterial catheter containing saline solution with heparin was connected to pressure transducers and advanced into the LV to monitor MAP, LVSP and LVEDP. All data were processed using the PowerLab system (AD Instruments, Sydney, Australia).

### HRV measurement

Electrocardiograms (ECGs) were obtained using the Powerlab system. Frequency domain parameters were derived using power spectrum analysis with low-frequency (LF: 0.04–0.15 Hz) and high-frequency (HF: 0.15–0.40 Hz) expressed in normalized units, and the ratio of low-frequency and high-frequency. Time domain analyses were evaluated using the SDNN intervals.

### Baroreflex sensitivity measurement

BRS testing was performed according to methods described previously^44^. After 30 min of baseline recordings, baroreflexes were elicited by bolus injection of phenylephrine (5 μg/kg, i.v.) through the right femoral vein. Each R-R interval was plotted as a function of the preceding systolic blood pressure. BRS was calculated as the slope of the linear portion of the relationship between the R-R interval (in ms) and the systolic arterial pressure (in mmHg). The results were included in the study only for animals with a correlation coefficient *R* > 0.8 and a *P* value < 0.05.

### Ang II Measurements

After hemodynamic studies, 2 mL blood samples were collected and centrifuged for 15 min (2000 *g* at 4 °C), and serum were stored at −80 °C. LV tissues were homogenized on ice in 0.9% saline and centrifuged for 15 min (12000 *g* at 4 °C) and stored at −80 °C. Ang II levels in serum and cardiac tissue homogenates was determined by radioimmunoassay using a commercially available kit (Bei Fang Radioimmunology Institute, Beijing, China) following the manufacturer’s instructions.

### Histological analyses

Collagen deposition in rat hearts was quantified using Masson’s trichrome staining and Picrosirius Red staining. Hearts were excised, washed in PBS at 4 °C, cut into 3 transverse sections close to the apex to visualize the left and right ventricles, and then fixed in 4% formalin for paraffin embedding. They were sectioned (5 μm) and stained. Cardiac fibrosis was analyzed using morphometry and visualized using light microscopy.

### Gelatin zymography and hydroxyproline measurement

Twenty microliters of culture supernatant from CFs with different treatments were electrophoresed in 10% SDS-PAGE containing 1% gelatin as MMP substrate under non-reducing conditions as described earlier^45^. After electrophoresis, the gel was washed in 1% Triton X-100 for 1 h. They were then incubated overnight at 37 °C in substrate buffer containing 0.05 M Trizma hydrochloride and 0.005 M Calcium chloride dihydrate (pH 8.0). The gel was stained in 0.1% Coomassie Blue R-250 and destained using 7.5% Acetic Acid and 5% Methanol until bands were clear. The gel was photographed and the active form of MMP-2 quantified using the Alpha Innotech gel capture system.

The content of hydroxyproline in CFs were assayed by using a commercial kit (Nanjing Jiancheng Bioengineering Institute, Nanjing, China) according to the manufacturer’s instructions.

### Assay for 5-ethynyl-2-deoxyuridine (EdU) incorporation

Cell proliferation was quantified by EdU incorporation into DNA using a Cell-Light TM EdU DNA Cell Proliferation Kit (RIBOBIO, Guangzhou, China). Briefly, cells were cultured in 24-well plates and exposed to 50 μM EdU for 4 h at 37 °C. Cells were then fixed in 95% ethanol for 30 min and permeabilized in 0.3% Triton X-100 for 10 min. Cells were washed with PBS and each well was incubated with 400 μL 1 × Apollo reaction cocktail for 30 min. DNA was then stained with 1 μg/mL PI (200 μL per well) for 10 min and imaged under a fluorescent microscope. Cell proliferation rate was calculated as the percentage of EdU-positive nuclei to total nuclei in five high-power fields per well.

### Cell migration

CFs migration was quantified using a transwell insert having 8-μm pore polyethylene terephthalate membranes as previously described^46^. Cells were serum-starved for 6 h, and 2.0 × 10^5^ cells/mL were seeded in the upper compartment. Cells were stimulated with Ang II (10^−7^ M). Plates were incubated at 37 °C for 24 h. Membranes were washed with PBS, and non-invading cells on the upper surface were removed using cotton swabs. Cells invading into and through the membranes were stained with 1 μg/mL PI and counted with a microscope at 200× magnification. Five fields were counted on each membrane. Migration is expressed as the mean value of total number of migrated cells per field.

### Immunofluorescence

Cells were fixed in 95% ethanol and permeabilized in 0.3% Triton X-100 in PBS. Cells were stained with anti-α-SMA antibody (1:100), anti-I collagen (1:200, Abcam), and anti-Smad3 (1:100) at 4 °C overnight, followed by incubation with a FITC anti-rabbit second antibody (1:200) and 1 μg/mL PI for 10 min at room temperature. Images were acquired by confocal microscopy.

### Western blot analysis

LV tissues and Cells proteins were extracted with a protease inhibitor cocktail-containing lysis buffer. Samples were resolved on 10% SDS-PAGE, transferred to a nitrocellulose membrane, and incubated with the following primary antibodies: anti-TGF-β1 (1:1000), anti-Smad3 (1:1000), anti-P-Smad3 (1:1000), anti-I collagen (1:500), anti-III collagen (1:500), and anti-MMP-2 (1:1000). All of them incubated at 4 °C overnight. The membranes were then incubated with IRDye-labeled secondary antibodies for 40 min (680RD Conjugated Goat polyclonal Anti-Mouse IgG, 1:4000, 800CW Conjugated Goat polyclonal Anti-Rabbit IgG, 1:4000). The membranes were scanned with the Odyssey Infrared Imager (LiCor Biosciences, Lincoln, NE, USA).

### Small interfering RNA transfection

CFs were cultured to 80% confluence and transfected with small interfering RNA (siRNA) of interest by using Lipofectamine 2000 (TurboFect, Themo Fisher Scientific). siRNA targeting M_2_ AChR and non-specific control siRNA were from GenePharma (Shanghai, China). After 48 h of transfection, cells were harvested and analyzed using western blot to confirm M_2_ AChR knockdown.

### Statistical analysis

All results are expressed as means ± SEM. *P* < 0.05 was considered statistically significant. Comparisons of results were performed by using one-way ANOVA analysis of variance followed by a Turkey post hoc test for multiple comparisons test using GraphPad Prism Version5.01 (GraphPad Software Inc, La Jolla, CA, USA).

## Additional Information

**How to cite this article**: Liu, J.-J. *et al.* Improving vagal activity ameliorates cardiac fibrosis induced by angiotensin II: *in vivo* and *in vitro*. *Sci. Rep.* **5**, 17108; doi: 10.1038/srep17108 (2015).
